# Finite element analysis of sacral fixation strategies for fragility fractures of the pelvis

**DOI:** 10.1038/s41598-026-45149-1

**Published:** 2026-03-20

**Authors:** Shenghong Liu, Libao Zhang, Changhui Xue, Chenwu Lu, Wanchen Gong, Zhengyi Lin, Min Li, Linfeng Wang

**Affiliations:** 1 Department of Orthopedics, The Affiliated Nanping First Hospital of Fujian Medical University, Nanping, Fujian China; 2 Department of Orthopedics, Jian’ou Municipal Hospital of Fujian Province, Jian’ou, Fujian China

**Keywords:** Fragility fracture of the pelvis, Sacroiliac screw, Sacral fracture, Finite element analysis, Anatomy, Diseases, Health care, Medical research

## Abstract

This study aimed to evaluate the biomechanical performance of different sacroiliac screw fixation strategies for posterior pelvic ring injuries in older adults with fragility fractures of the pelvis. A finite element model was created using the pelvis of an older woman with combined anterior and posterior ring injuries, simulating a unilateral pubic rami fracture and a Denis zone I sacral fracture. A subcutaneous internal fixator (INFIX) system was used to support the anterior pelvic ring. Percutaneous sacroiliac screws of different lengths and fixation levels were used to create six posterior fixation configurations. The peak von Mises stress within the INFIX system remained below 4 MPa across all configurations, whereas the maximum displacement at the pubic fracture site was < 0.04 mm. Among posterior constructs, the dual-segment long screw configuration showed the lowest sacral fracture displacement (0.02 mm) and the highest screw stress (28.66 MPa). Compared with single-level fixation, constructs with both S1 and S2 fixation demonstrated less fracture displacement and superior load distribution patterns. Furthermore, compared with short screws, long screws exhibited distinct load-sharing features, suggesting improved stress transfer through the posterior pelvic ring. In conclusion, dual-segment sacroiliac screw fixation—particularly using long trans-iliac–trans-sacral screws spanning both S1 and S2 levels—provided improved fracture stability and more advantageous load-sharing behavior in this simulation setting, both in the osteoporotic finite element model and under static, symmetric loading conditions.

## Introduction

The incidence of osteoporosis-related fragility fractures of the pelvis (FFP) continues to rise among older adults because of the consistent increase in life expectancy worldwide^1–3^. According to epidemiological data, the annual incidence among those over 65 years is approximately 92 per 100,000, and among those aged 85 years and older, it reaches up to 446 per 100,000. It is predicted that this tendency will continue to rise over the next 10 years^4^. FFP is frequently associated with pneumonia, deep vein thrombosis, delirium, and functional decline from protracted immobility, which places a substantial burden on patients and healthcare systems. Thus, achieving stable fixation and promoting early mobilization—while minimizing perioperative risks—has emerged as a pressing clinical issue.

Older adults often present with multiple chronic comorbidities and severe osteoporosis, which makes them less responsive to traditional open surgery. In such patients, intraoperative blood loss, extensive soft tissue injury, and delayed postoperative recovery are particularly concerning. In contrast, by minimizing soft tissue damage and blood loss, facilitating early mobilization and rehabilitation, and improving general quality of life, minimally invasive fixation techniques have demonstrated distinct benefits for treating pelvic fractures^5–7^. Percutaneous sacroiliac screw fixation has emerged as the most popular posterior stabilization procedure for stabilizing posterior pelvic ring injuries owing to its minimally invasive design^8^.

Sacral fracture fixation is crucial for preserving pelvic ring stability and ensuring load transfer during early mobilization. However, there remains disagreement over the “optimal” sacroiliac screw configuration for osteoporotic posterior pelvic ring injuries, particularly regarding the screw length and fixation level^8–12^. These factors affect both early postoperative weight-bearing capacity and the probability of internal fixation failure by influencing load distribution and rotational stability at the bone-implant interface^12–14^. Finite element analysis (FEA) offers a strong computational framework to methodically assess the biomechanical effects of alternative fixation strategies, considering the ethical and real-world challenges of performing a randomized controlled trial in this population.

This study focused on a combined injury pattern that is prevalent in older adults^9,15^. A right-sided pelvic fracture with anterior (superior and inferior pubic rami) and posterior (Denis zone I sacral fracture) rings was simulated using an FE model. Prior finite element research has mostly focused on posterior ring fixation in isolation^12,16–18^. The biomechanical interaction between anterior and posterior structures may affect load distribution, and fragility fractures usually entail both anterior and posterior ring instability. Nonetheless, sacroiliac screw configurations within a complete anterior–posterior fragility fracture construct under standardized osteoporotic conditions have not been thoroughly investigated. Multiple sacroiliac screw fixation procedures, which varied by screw length and fixation level, were applied to the posterior ring under standardized anterior ring fixation using the subcutaneous anterior pelvic bridge (INFIX) system^7^. In this study, an integrated pelvic ring model with anterior INFIX stabilization was used to evaluate the mechanical performance of each sacroiliac screw configuration under standardized loading conditions, facilitating construct-level comparison within a complete pelvic ring framework. To optimize customized fixation techniques in clinical practice, this study aimed to provide quantitative biomechanical evidence to direct posterior ring fixation strategies in older adults with FFP. This study aims to fill a substantial gap in the biomechanical evaluation of osteoporotic fragility fracture repair by integrating anterior and posterior stabilization, incorporating element-based stress evaluation, and allowing for construct-level comparison under uniform loading circumstances. These findings might help improve fixation selection in patients undergoing minimally invasive surgery for osteoporotic fractures.

## Results

### Model validation

**Fig. 1:**
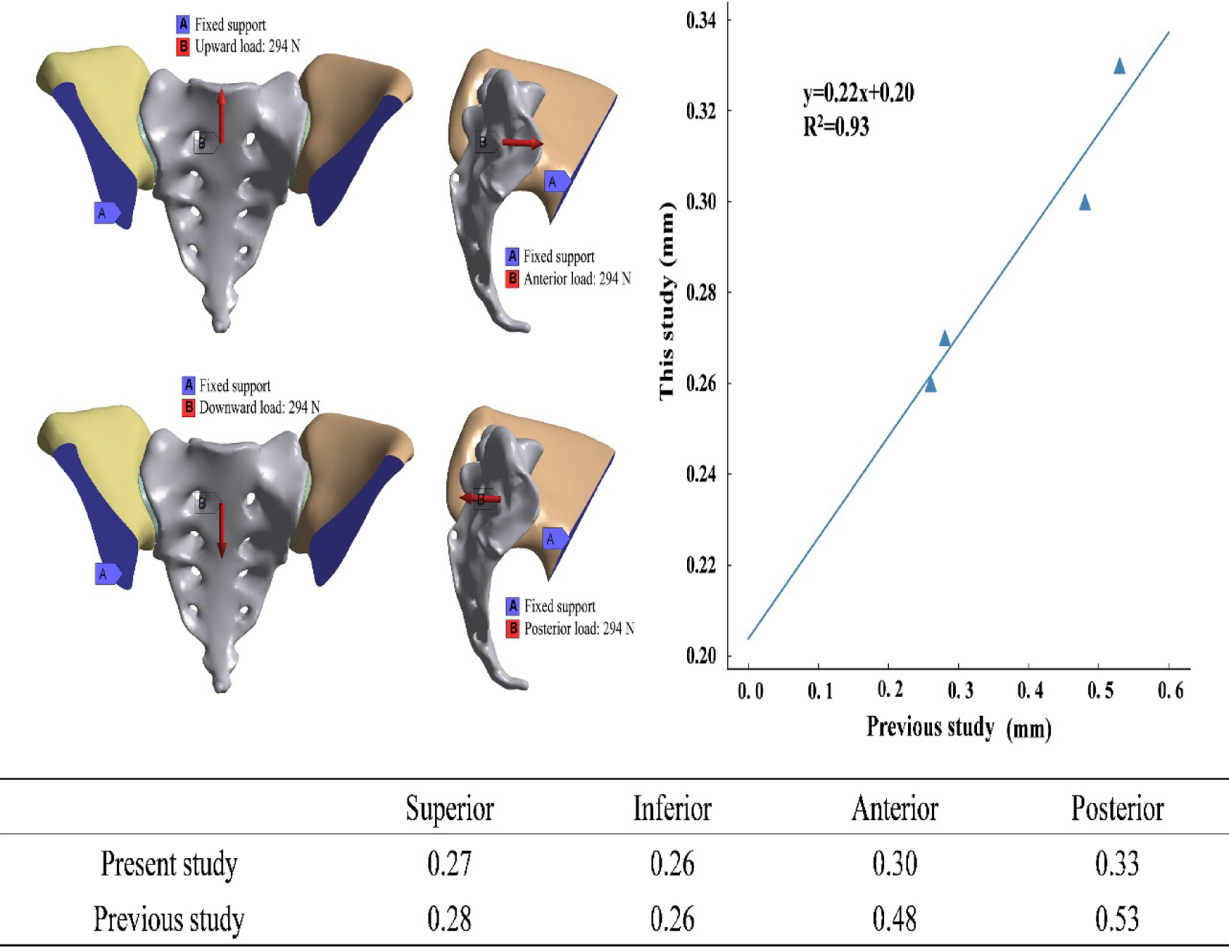
Validation of the finite element pelvic model. The left panel shows four unidirectional loading conditions for validation: upward, downward, anterior, and posterior loads of 294 N applied at the sacrum center while both iliac bones are fixed. The right panel shows the consistency analysis between the validation and reference data (Pearson correlation, R² = 0.93), indicating a high degree of agreement.

To evaluate the reliability of the FE model, a validation protocol adapted from a previously published loading scheme^19^ was used. A unidirectional load of 294 N was applied successively at the sacral center in the superior, inferior, anterior, and posterior directions while both iliac bones were restrained to maintain stability. To assess mechanical consistency, the corresponding sacral center displacements under each loading direction were extracted and compared with reference data. A strong linear correlation between the displacements predicted using FE analysis and the reference values confirmed the pelvic model’s mechanical repeatability and external validity (Fig. 1). These findings support the model’s applicability for comparative investigation of different fixation techniques.

### Mechanical performance of anterior ring fixation

**Fig. 2:**
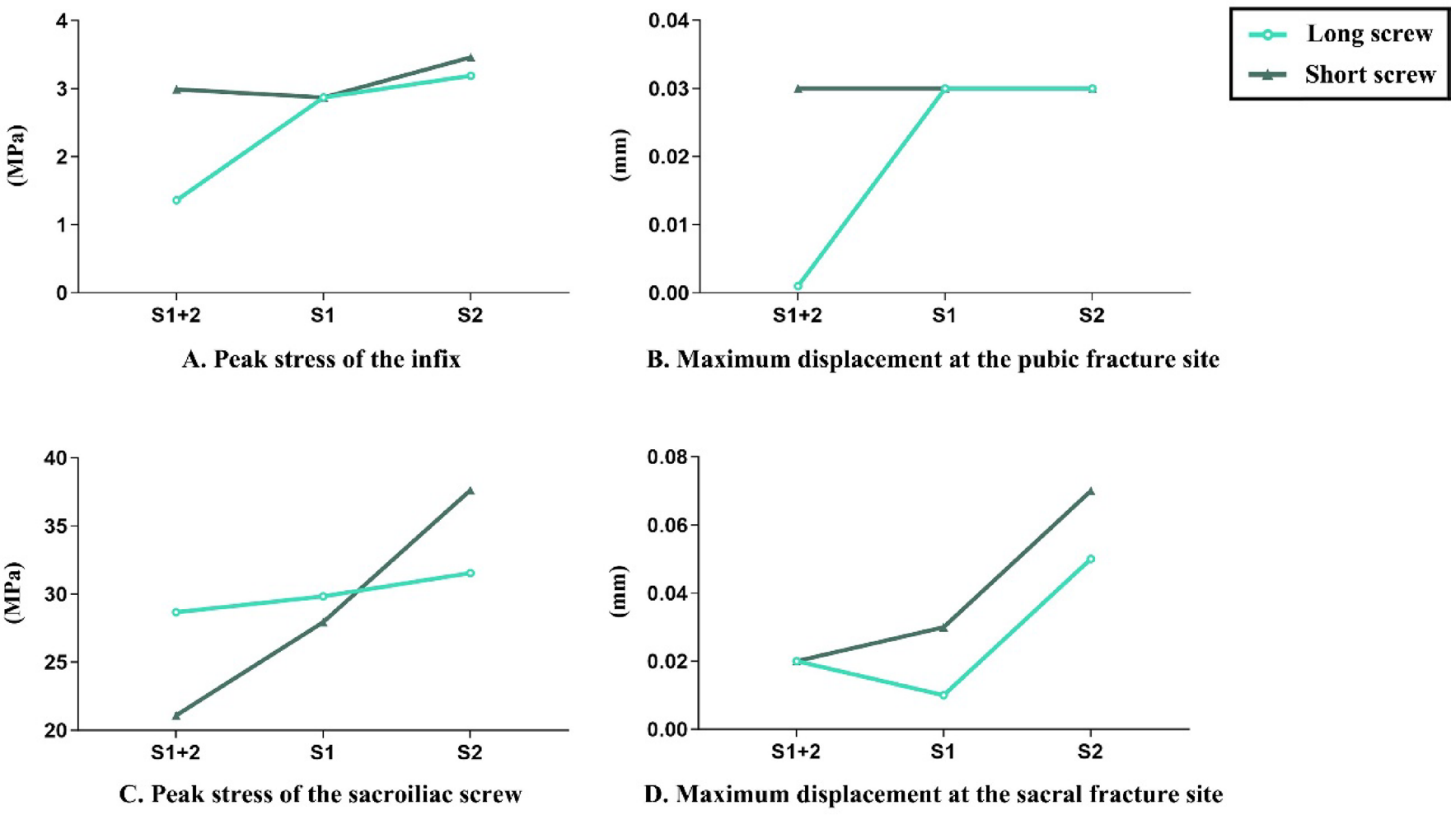
Finite element analysis results of the six models.

The peak von Mises stress within the INFIX system remained below 4 MPa across all six fixation configurations, and the maximum displacement at the pubic fracture site was < 0.04 mm (Figs. 2A, and B). Therefore, under the imposed loading conditions, the anterior ring fixation consistently offered satisfactory biomechanical support. The SS2 model showed the highest peak stress inside the INFIX system, whereas the LS1 + 2 configuration showed the lowest peak stress and the least pubic fracture displacement (Fig. 2A and B). Consequently, a lower INFIX stress may be associated with a decreased risk of implant fatigue or long-term mechanical failure. Additionally, peak INFIX stress and maximal pubic fracture displacement were comparable between long- and short-screw constructs under single-segment posterior fixation. Thus, screw length at a single fixation level did not considerably change anterior ring mechanics within this modeling framework.

**Fig. 3:**
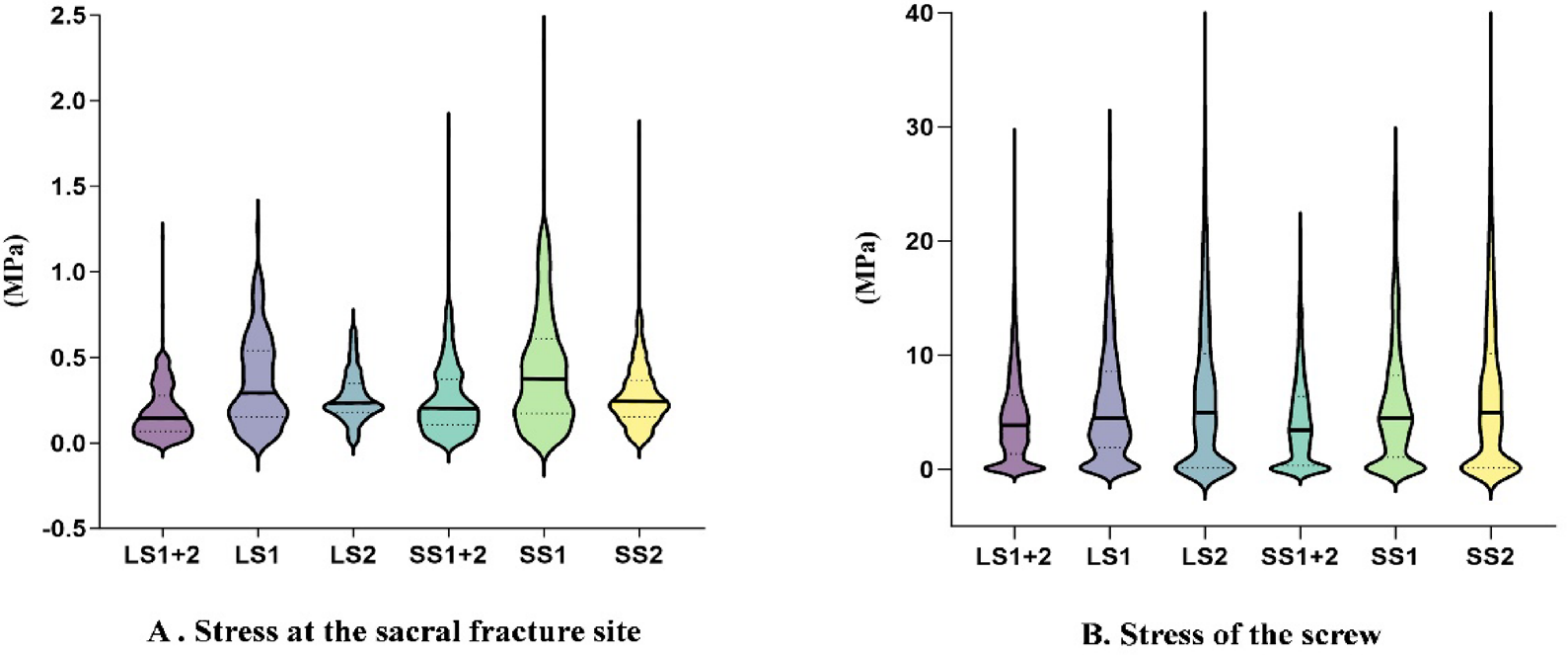
Comparative full-field stress distribution among the six finite element models.

**Fig. 4:**
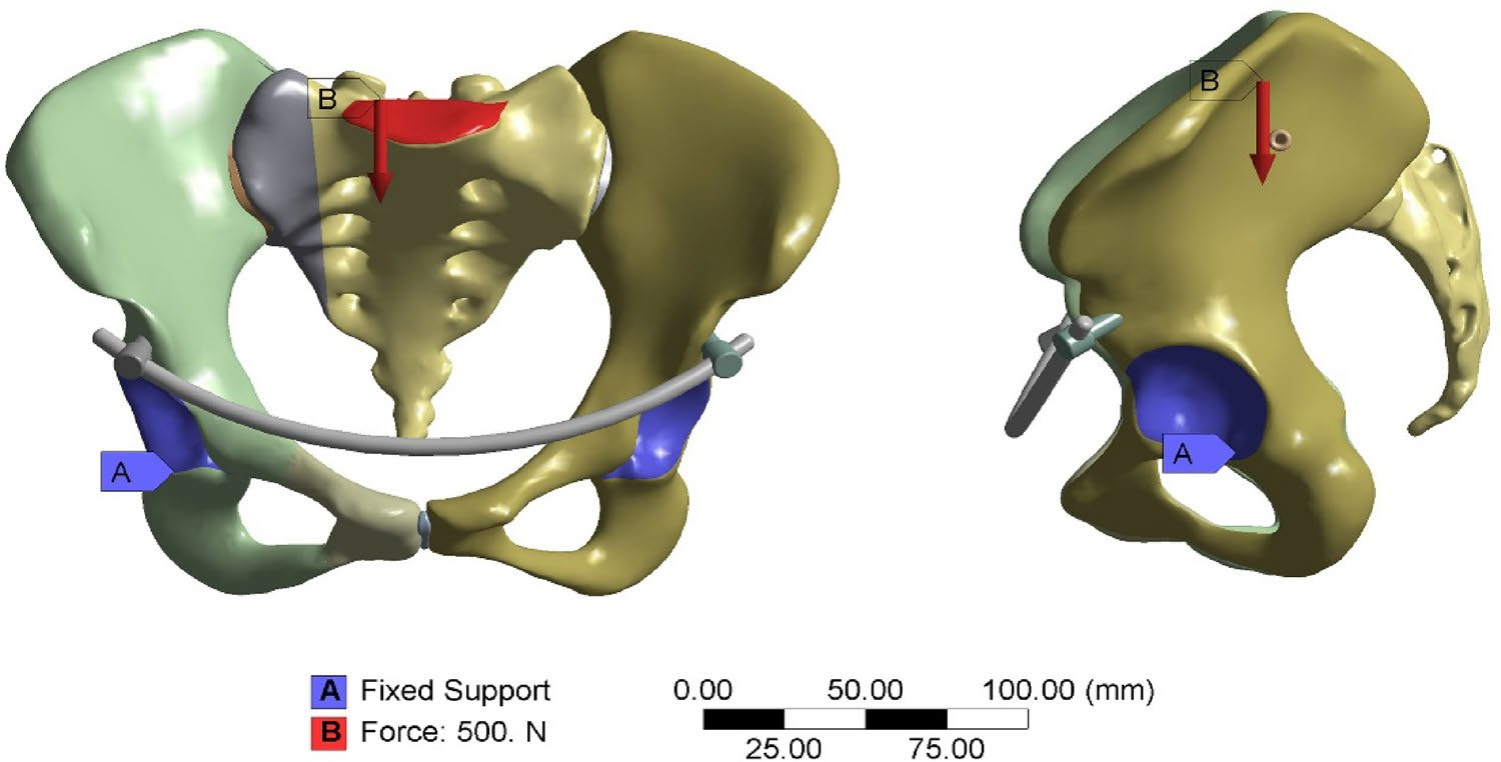
Fracture model, boundary conditions, and loading. Fracture pattern: Anterior pelvic ring fracture involving both superior and inferior pubic rami in conjunction with a Zone I sacral fracture in the posterior ring. Boundary conditions: Inner walls of both acetabula are constrained in the x, y, and z directions. Loading: The superior endplate of S1 receives a uniformly distributed vertical load of 500 N applied downward.

### Mechanical performance of posterior ring fixation

Peak von Mises stress within the sacroiliac screws showed identical distribution patterns for both long-screw and short-screw constructs (Fig. 2C). Peak stress levels were highest in the LS2 and SS2 configurations and lowest in the LS1 + 2 and SS1 + 2 configurations. Interestingly, the SS1 + 2 configuration showed the lowest peak stress in the sacroiliac screws, whereas the SS2 configuration showed the highest peak stress in the iliac screws. Variations in peak screw stress across three long-screw configurations were comparatively small. In sacroiliac screws, the following pattern was observed: LS1 + 2 > SS1 + 2, LS1 > SS1, and LS2 < SS2. With values comparable to those observed in LS1 + 2 and SS1 + 2 configurations, the LS1 configuration showed the smallest sacral fracture displacement. Conversely, the SS2 configuration showed the largest displacement at the sacral fracture site, indicating worse construct stability (Fig. 2D).

### Element-based stress distribution analysis

Element-based analysis of full-field von Mises stress was conducted on both the sacral fracture surface and the sacroiliac screws to reduce the impact of localized numerical singularities. Across both long- and short-screw constructs, the sacral fracture surface demonstrated a consistent stress pattern, with single-segment configurations (S1 and S2) exhibiting higher stress values than the dual-segment configuration (S1 + 2) (Fig. 3A). Thus, the S1 + 2 configuration was associated with decreased stress distribution at the fracture interface under the simulated loading conditions. In cross-group comparison, the LS1 + 2 construct demonstrated lower fracture surface stress values than the SS1 + 2 construct in the model output, indicating improved load-sharing. Both the long- and short-screw groups showed a comparable intra-group pattern in screw stress distribution: within each group, single-segment configurations (S1 or S2) had higher screw stress than the dual-segment configuration (S1 + 2). Thus, dual-segment fixation may improve stress redistribution across the screws within this simulation. However, when comparing long- and short-screw constructs, the LS1 + 2 construct demonstrated higher screw stress values than the SS1 + 2 construct, whereas the LS1 construct showed higher stress than the SS1 construct (Fig. 3B).

## Discussion

In older adults with FFP, minimally invasive fixation techniques are essential for reducing intraoperative blood loss, minimizing surgical trauma, and promoting postoperative recovery. Notably, FEA was used in this study to assess sacral fixation techniques for osteoporotic pelvic fractures under standardized modeling and loading conditions. Compared with dual-segment (S1 + 2) fixation, single-screw fixation–either at the S1 or S2 level–demonstrated higher implant displacement and stress values, suggesting worse mechanical performance regarding stress redistribution and stability.

Under the applied axial loading conditions, all six sacral fixation configurations demonstrated high anterior ring stability when paired with anterior stabilization using the INFIX system. Therefore, INFIX provides sufficient mechanical support for anterior ring stabilization in this simulated condition. The LS1 + 2 configuration (long trans-iliac–trans-sacral screws at both S1 and S2 levels) demonstrated lower fracture displacement and implant stress among the evaluated constructs, whereas the SS2 configuration (short screw at the S2 level) exhibited higher displacement and stress values. Additionally, the maximal pubic fracture displacement and peak INFIX stress were similar for long- and short-screw designs under single-segment posterior fixation. Thus, screw length alone may have a limited impact on anterior ring load distribution and overall stability upon using only one fixation level (S1 or S2).

Regarding posterior ring fixation, the S1 + 2 dual-segment approach was associated with decreased sacroiliac screw stress and sacral fracture displacement, suggesting a more favorable mechanical distribution within the simulated construct. Additionally, sacroiliac screws followed the pattern of LS1 + 2 > SS1 + 2 and LS1 > SS1, whereas LS2 < SS2. These stress patterns indicated a level-dependent interaction between fixation level and screw length. Therefore, the screw length interacts with sacral-level-specific load-bearing qualities rather than producing a uniform biomechanical effect. Under single-segment fixation, S1 serves as the primary load-bearing level of the posterior ring; therefore, a trans-sacral long screw creates a spanning construct with a longer lever arm and higher constraint rigidity. In addition to axial loading, this configuration may result in increased bending and shear components inside the implants, increasing the peak von Mises stress within the screw. In contrast, a long trans-sacral screw at S2 may improve load redistribution and reduce localized stress concentration, resulting in a lower peak stress than a short-screw construct. This is because S2 is typically a secondary load-bearing level.

In dual-segment fixation, the use of two long screws increases global posterior ring stiffness and improves fracture stability. However, it may increase peak screw stress by simultaneously transferring a larger percentage of load to the implants. This phenomenon reflects the bio-mechanical trade-off between enhanced construct stability and increased implant stress demand. The LS1 + 2 configuration showed a non-monotonic trend concerning maximal displacement at the sacral fracture site (Fig. 4D). We hypothesized that the inclusion of a second fixation level changed the posterior ring stiffness distribution and constraint coupling patterns, after LS1 alone provided sufficient local constraint. Thus, the marginal benefit of dual-segment fixation was more evident in global stiffness improvement and load-sharing optimization rather than in a strictly linear reduction of a single peak displacement parameter. Importantly, this does not imply decreased overall stability; rather, it indicates that the additional segment primarily improved global stiffness and load redistribution after effective local displacement control by LS1.

Moreover, the element-based full-field stress analysis showed that the S1 + 2 configuration consistently reduced stress concentrations at the sacral fracture surface as well as within the sacroiliac screws. Under the specified modeling assumptions, these findings emphasize a relative mechanical advantage in load redistribution. The LS1 + 2 configuration transferred a higher percentage of load to the screws than SS1 + 2, which reduced stress concentration at the fracture interface and provided a more stable mechanical environment for fracture healing. However, this advantage should not be immediately generalized to predict clinical healing outcomes or long-term fixation durability, because it reflects only a relative trend under the simulated conditions.

From a clinical perspective, less fracture displacement may contribute to improved early mechanical stability and a biomechanically conducive environment for callus development. In contrast, elevated screw stress may raise fatigue demand and the risk of loosening, particularly in osteoporotic bone with decreased cortical purchase. However, in the present model, all peak stress values remained below the yield strength of the titanium alloy, indicating that the differences are attributed to relative construct-level mechanical changes rather than imminent implant failure. Therefore, these findings should be interpreted as comparative construct-level biomechanics rather than direct predictors of postoperative complications or anatomic outcomes.

The mechanical patterns observed in this study are consistent with previous research showing the biomechanical implications of trans-sacral fixation or dual-level fixation techniques for pelvic stability. For example, Eckardt et al. found that using S1 or S2 fixation alone increased the likelihood of internal fixation failure in their study of 50 clinical cases of pelvic fragility fractures. Additionally, compared with short screws, the use of trans-iliac trans-sacral (TITS) screws yielded lower reoperation rates^12^. For bilateral zone II sacral fractures, Zhou et al.^16^ investigated eight sacroiliac screw configurations, including standard, long, and TITS screws. Dual-segment fixation and double TITS screw constructs demonstrated superior mechanical performance. In an FE analysis, Zhao et al.^17^ reported that longer screws exhibited superior stability; they suggested using two trans-sacral screws at the S1 and S2 levels. Furthermore, several cadaveric studies have confirmed that two-screw constructs offer substantially greater stability than single-screw fixation^18^. Taken together, these studies support the biomechanical rationale for dual-segment fixation with long screws; however, cautious translation to clinical practice is warranted in older adults with pelvic fragility fractures.

Previous studies have elucidated the biomechanical advantages of dual-segment or trans-sacral fixation. Nonetheless, the current study adds to the body of knowledge by evaluating these constructs within an integrated anterior-posterior fragility fracture model with unified osteoporotic parameters. Instead of evaluating posterior fixation alone, this study offers a construct-level biomechanical comparison by conducting element-based full-field stress analysis and integrating anterior stabilization utilizing the INFIX system. This integrated framework allows for a more comprehensive understanding of load-sharing behavior and stability patterns in older adults with pelvic fragility fractures, offering improved mechanistic insights into fixation strategy optimization.

Dual-segment fixation reduced the peak von Mises stress of sacroiliac screws (Figs. 2C and 3B). This finding may reduce the risk of mechanical failure, such as implant loosening or fatigue under conditions of repetitive loading^16^. This issue is particularly important in vertically unstable sacral fractures, where repeated axial and shear stresses may accelerate implant stress accumulation^20,21^. Additionally, this configuration reduced fracture displacement (Fig. 2D) and stress concentrations at the sacral fracture surface (Fig. 3A), suggesting a mechanically favorable environment for early stability within the constraints of the current model.

Because long screws pass through both sacroiliac joints and engage the bilateral iliac cortices, they increase the working length and promote more uniform load distribution. Hence, they demonstrated mechanical advantages in this simulation. By lowering local stress peaks, this configuration promoted superior vertical and rotational stability under the simulated axial loading situation. However, the biomechanical advantages must be carefully weighed against anatomical and technical issues that arise in clinical practice. Sacral nerve root irritation and vascular compromise are among the possible neurovascular damage risks associated with trans-sacral screw insertion. Sacral dysmorphism, which is common in older adults, may substantially limit the safe osseous corridor for screw insertion. Furthermore, reduced osteoporotic bone quality and fluoroscopic limitations may increase technical difficulty and the risk of malposition^22^. Under such circumstances, dual-segment fixation with short screws is still a viable alternative because it minimizes the risk of cortical rupture or neural damage while preserving a satisfactory level of mechanical stability. Because single-segment fixation showed higher stress and displacement values in the current model, its clinical application should be carefully considered, particularly in osteoporotic bone.

This study has several limitations. First, modeling cortical and cancellous bone as linear, homogeneous, and isotropic materials may not accurately reflect the anisotropic and heterogeneous properties of osteoporotic bone. This assumption is frequently adopted in comparative FE studies; nonetheless, it may influence absolute stress magnitudes. Second, the use of linear fracture lines facilitated the controlled evaluation of fracture interface mechanics; however, the irregular, comminuted morphology typical of clinical sacral fractures was not entirely replicated. Third, this model did not account for interindividual variations in sacral shape or bone mineral density because it was derived from a single anatomical dataset. Therefore, the findings are based on deterministic biomechanical comparisons rather than population-based statistical inference. Additionally, fully bonded contacts were used to define screw–bone interfaces, which may not reflect micro-motion or progressive loosening observed in clinical settings. The introduction of a static 500 N vertical load in conjunction with complete acetabular constraint represents a simplified boundary condition that may affect load transfer patterns. Different loading scenarios, such as unilateral stance, torsional loading, or partial weight bearing, may alter the posterior load distribution and the relative ranking of fixation constructs. Furthermore, using different anterior anchoring techniques may result in different posterior construct behavior. Finally, before wider clinical application can be justified, findings from this 3D FE model warrant further validation through experimental, cadaveric, and clinical studies.

## Methods

### Materials

After excluding patients with a history of pelvic deformities, previous fractures, or bone tumors, one older woman (65 years old, 158 cm tall, and 55 kg in weight) who underwent a complete abdominal computed tomography (CT) was selected. The patient had no history of pelvic trauma or surgery. A scanner set with a slice thickness of 0.625 mm and settings of 120 kV and 500 mA was used to obtain pelvic CT images. The study was approved by the institutional ethics committee (Approval No. NPSY202506039), and informed consent was obtained from the patient.

### Methods

Mimics Research 21.0 (Materialise, Leuven, Belgium) was used to import the original Digital Imaging and Communications in Medicine data. Seed points were positioned on the iliac cortical bone using the ADVANCED SEGMENT – CT Bone workflow, and threshold growth was automatically applied until the mask covered the entire pelvis. Bilateral hip bones, the sacrum, and femurs were segmented and removed separately. The “Calculate Part” function was used to create 3D solid models with optimal quality. For geometric optimization, which included surface smoothing, hole filling, and the elimination of artifacts or spike-like features, the generated STL files were exported to Geomagic Wrap 2021 (Hexagon AB, Sweden). The cortical bone contours that resulted from this procedure were the continuous and smooth outer surfaces of the sacrum and bilateral hip bones. After reconstructing and meshing the cortical surfaces in a precise surface mode, the files were exported in STP format.

Using the offset tool, the cortical contours were offset inward to create the cancellous bone geometry, which considered regional differences in cortical bone thickness. The complete pelvic model was assembled using entity duplication and Boolean operations after importing the cortical and cancellous bone geometries into SOLIDWORKS 2023 (Dassault Systèmes).

**Fig. 5:**
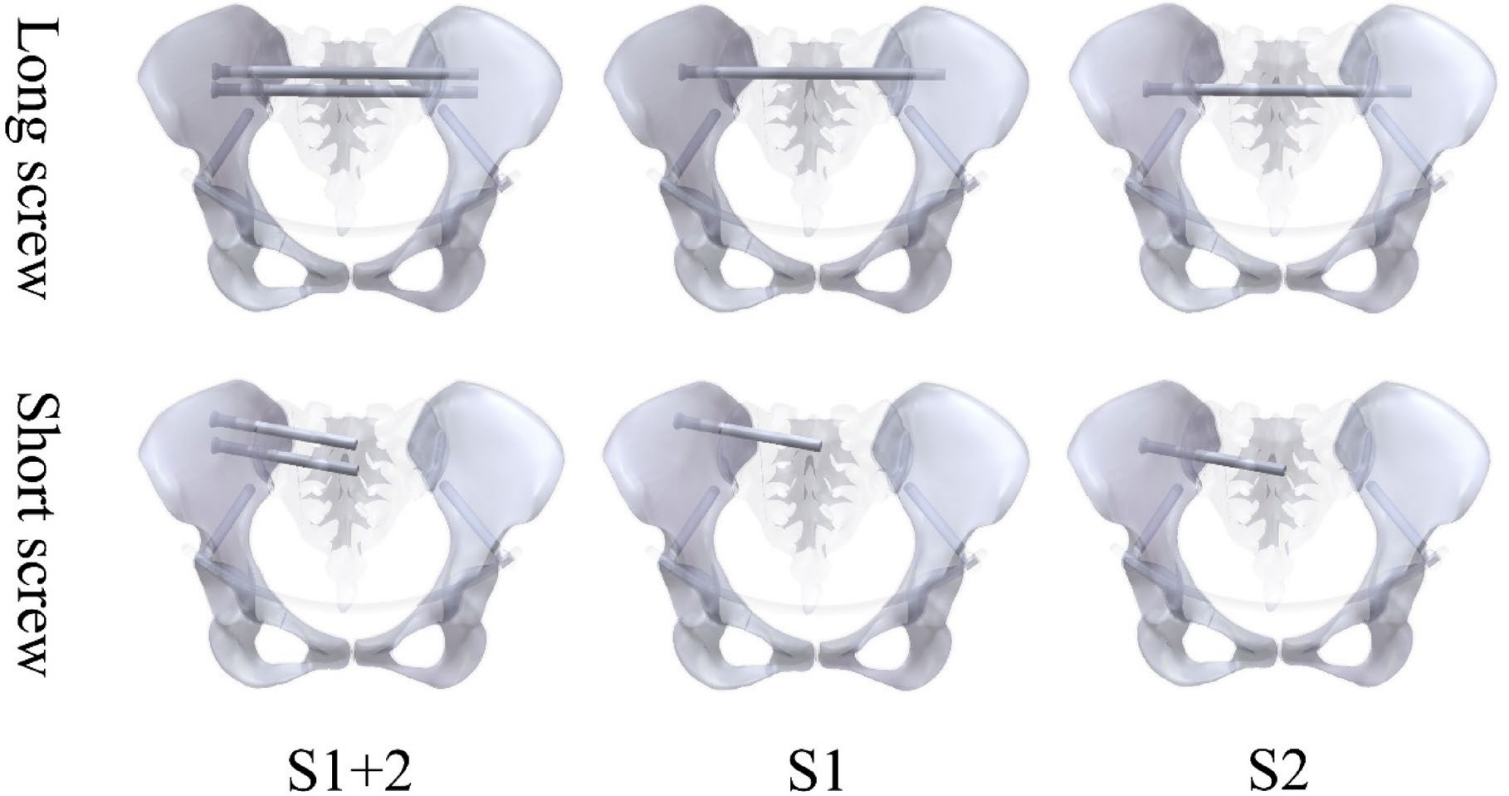
Grouping of the finite element models. The sacroiliac screws are modeled as full-thread screws with a diameter of 7.3 mm.

A composite injury model was created using segmentation tools to simulate fractures of the superior and inferior pubic rami (anterior ring) and a Denis zone I sacral fracture (posterior ring) based on published fracture patterns (Fig. 4)^15^. Parametric models of the INFIX and percutaneous sacroiliac screws—each with varying lengths and fixation levels—were constructed in SOLIDWORKS 2023 and meticulously assembled using the pelvic model, which was tailored to the patient’s pelvic anatomy. Variations in posterior ring fixation strategies led to the development of six FE models (Table 1; Fig. 5).

**Table 1:** Posterior ring fixation configurations of the six models.

Group	Anterior ring fixation	Posterior ring fixation	Posterior fixation level
LS1	INFIX	One long screw	S1
LS2		One long screw	S2
LS1 + 2		Two long screws	S1 + S2
SS1		One short screw	S1
SS2		One short screw	S2
SS1 + 2		Two short screws	S1 + S2

The short screw refers to the standard sacroiliac screw, whereas the long screw refers to the trans-iliac–trans-sacral screw. In the finite element model, all screws were modeled as full-thread titanium alloy screws with a diameter of 7.3 mm and were geometrically simplified as smooth cylinders.

For FE analysis, all six rebuilt pelvic models were imported into ANSYS Workbench 2022R1 (ANSYS, USA). The values reported for osteoporotic populations were used to assign attributes to the bone material. Table 2 summarizes the corresponding elastic moduli and Poisson’s ratios^23,24^. To facilitate computational convergence and ensure intergroup comparability, both osseous structures and metallic implants were modeled as linear, homogeneous, and isotropic elastic materials. Major pelvic ligaments were simplified as tension-only spring elements, and axial stiffness values were derived from previously published biomechanical data (Table 3)^25^. To simulate ideal fixation, contact interactions were defined as follows: (i) threaded screw–bone interfaces were modeled as bonded; (ii) non-threaded bone–implant interfaces were assigned a friction coefficient of 0.2; and (iii) fracture surfaces were modeled using surface-to-surface contact with a friction coefficient of 0.3^26^.

Second-order tetrahedral elements were used to mesh all models. The maximal displacement at the sacral fracture site and the peak von Mises stress within the screws were used to evaluate mesh convergence. Mesh independence was confirmed by gradually decreasing the global initial mesh size of 4 mm until the relative change in these indicators was < 5%. After three iterative refinements, the final element sizes were determined to be 3 mm for bone structures and metallic implants and 2 mm for soft tissue and ligament attachment regions. Table 4 provides comprehensive mesh statistics.

**Table 2:** Material property assignments.

Material	Young’s modulus (MPa)	Poisson’s ratio
Cortical bone	11,390	0.3
Cancellous bone	33	0.2
Titanium alloy	110,000	0.3
Sacroiliac joint	10	0.4
Pubic symphysis	5	0.45

**Table 3:** Parameters of sacroiliac joint–related ligaments.

Ligament	Stiffness(*N*/mm)	Number
Anterior sacroiliac ligament	700	24
Posterior sacroiliac ligament	1400	16
Sacrospinous ligament	1400	12
Sacrotuberous ligament	1500	12
Superior pubic ligament	500	4
Arcuate pubic ligament	500	4
Interosseous ligament	2800	10

**Table 4:** Mesh division data of the six finite element models.

Model	Number of nodes	Number of elements
LS1	452,424	267,304
LS2	453,274	267,973
LS1 + 2	457,713	269,315
SS1	451,002	267,128
SS2	450,457	266,807
SS1 + 2	454,083	268,396

A homogenous 500 N vertically downward force was applied to the superior surface of the S1 vertebral body to replicate a physiological standing position. To simulate bilateral lower limb support, nodes on the inner surfaces of both acetabula were completely constricted in all translational degrees of freedom (x, y, and z axes) (Fig. 4)^24^. A static vertical load of 500 N was applied to provide a standardized and controlled loading framework for relative construct comparison, rather than to completely replicate complex dynamic physiological conditions. By reducing confounding biomechanical variables, this modelling approach allows for consistent evaluation of fixation configurations under identical mechanical conditions.

The following biomechanical outcome metrics were established to reduce numerical singularities at sharp corners and contact interfaces and to comprehensively assess fixation performance:

### Anterior ring indicators

1. Peak von Mises stress in the INFIX system;
2. Maximum displacement at the pubic fracture site.

### Posterior ring indicators

1. Peak von Mises stress within the sacroiliac screws;
2. Maximum displacement at the sacral fracture site;
3. Full-field stress distribution across the sacral fracture site;
4. Full-field stress distribution within the sacroiliac screws.

### Statistical analysis

The relationship between the FEA-predicted values and reference data was assessed using Pearson correlation analysis as part of the model validation process. IBM SPSS Statistics version 29.0.1.0 (IBM Corp., Armonk, NY, USA) was used for all statistical procedures.

## Conclusions

This study utilized finite element analysis to compare different sacral fixation configurations for pelvic fragility fractures in older adults. Dual-segment fixation at both S1 and S2 levels using long screws engaging the bilateral iliac cortices demonstrated lower fracture-site displacement and implant stress across multiple biomechanical indicators, under the standardized modeling assumptions and static loading conditions. Therefore, this configuration may improve the mechanical stability of the osteoporotic construct. In this computational framework, dual-segment structures that combined long and short screws or used two short screws showed acceptable biomechanical performance when patient-specific anatomical conditions prevented the safe placement of long trans-sacral screws. Therefore, when customized to each patient’s unique sacral shape and available corridor, these configurations may be considered as alternative surgical options.

## Data Availability

No datasets were generated or analyzed during the current study. The finite element models generated and analyzed during the current study shall be made available from the corresponding author upon reasonable request.
